# 

**Published:** 1922-01

**Authors:** 


### OUR PRIZE LEAFLET COMPETITION.

### EATING AND HEALTH.

THE RESULTS of this Competition were very disappointing. No doubt the short time at our competitors' disposal detracted from the efficiency of their performances—they had only four or five days in which to compose their leaflets. This was mostly the fault of the Christmas season, and we therefore do not apologise for it. One competitor, however, wrote blaming us, saying, "By the time that I had read up the Encyclopædia and discovered the awful end of various and notorious gluttons, it was too late to write you a leaflet; so I send you this letter of protest instead; for no one can be expected to write a leaflet on a subject like Eating and Health without the most careful consideration of the literature." We disagree; for we believe that the best leaflets are the work of a few moments and are written spontaneously without reference to vast volumes. But he will be given another chance.

We do not propose to print any of the leaflets received this month, nor do we award to anyone a prize. As we consider that the subject is worth a good leaflet we set this competition again; and we offer on this occasion a prize of *Five Guineas* for the best leaflet on Eating and Health. Those who competed last month may try again, and we hope that they will be successful in producing something more polished and instructive than we have already received from them.

### A Leaflet Competition

*We offer* **FIVE GUINEAS** *for the best new and original leaflet of not more than* 300 *words on*

**"EATING and HEALTH"**

The Leaflet must be suitable for circulation in the Public Elementary Schools, and among the Scholars of the Secondary Schools. The result of this Competition will be announced and the successful Leaflet will be printed in the February Number of "The Hospital and Health Review." We reserve the right to republish any of the Leaflets submitted to us.

RULES AND REGULATIONS.

1. This Competition is open to all.
2. Leaflets must be written or typed on unprinted paper on one side of the page only.
3. Competitors must employ a pseudonym.
4. Leaflets must be received by the Editor at "The Hospital and Health Review," 28 Southampton Street, Strand, W.C.2, by **first post on January 16, 1922.** Envelopes must be marked in the top left-hand corner with the word "Leaflet."
5. The Editor's decision is final.

